# The Biological Functions and Regulatory Mechanisms of Fatty Acid Binding Protein 5 in Various Diseases

**DOI:** 10.3389/fcell.2022.857919

**Published:** 2022-04-04

**Authors:** Binyue Xu, Lu Chen, Yu Zhan, Karl Nelson S. Marquez, Lvjia Zhuo, Shasha Qi, Jinyu Zhu, Ying He, Xudong Chen, Hao Zhang, Yingying Shen, Gongxing Chen, Jianzhong Gu, Yong Guo, Shuiping Liu, Tian Xie

**Affiliations:** ^1^ Department of Oncology, The First Affiliated Hospital of Zhejiang Chinese Medical University, Hangzhou, China; ^2^ School of Pharmacy, Hangzhou Normal University, Hangzhou, China; ^3^ Key Laboratory of Elemene Class Anti-Cancer Chinese Medicines, Engineering Laboratory of Development and Application of Traditional Chinese Medicines, Collaborative Innovation Center of Traditional Chinese Medicines of Zhejiang Province, Hangzhou Normal University, Hangzhou, China; ^4^ Clinical Medicine, Tongji Medical College, Huazhong University of Science and Technology, Hankou, China

**Keywords:** FABP5, lipid metabolism, lipid homeostasis, cell differentiation, immune response, tumorigenesis

## Abstract

In recent years, fatty acid binding protein 5 (FABP5), also known as fatty acid transporter, has been widely researched with the help of modern genetic technology. Emerging evidence suggests its critical role in regulating lipid transport, homeostasis, and metabolism. Its involvement in the pathogenesis of various diseases such as metabolic syndrome, skin diseases, cancer, and neurological diseases is the key to understanding the true nature of the protein. This makes FABP5 be a promising component for numerous clinical applications. This review has summarized the most recent advances in the research of FABP5 in modulating cellular processes, providing an in-depth analysis of the protein’s biological properties, biological functions, and mechanisms involved in various diseases. In addition, we have discussed the possibility of using FABP5 as a new diagnostic biomarker and therapeutic target for human diseases, shedding light on challenges facing future research.

## Introduction

**FIGURE 1:**
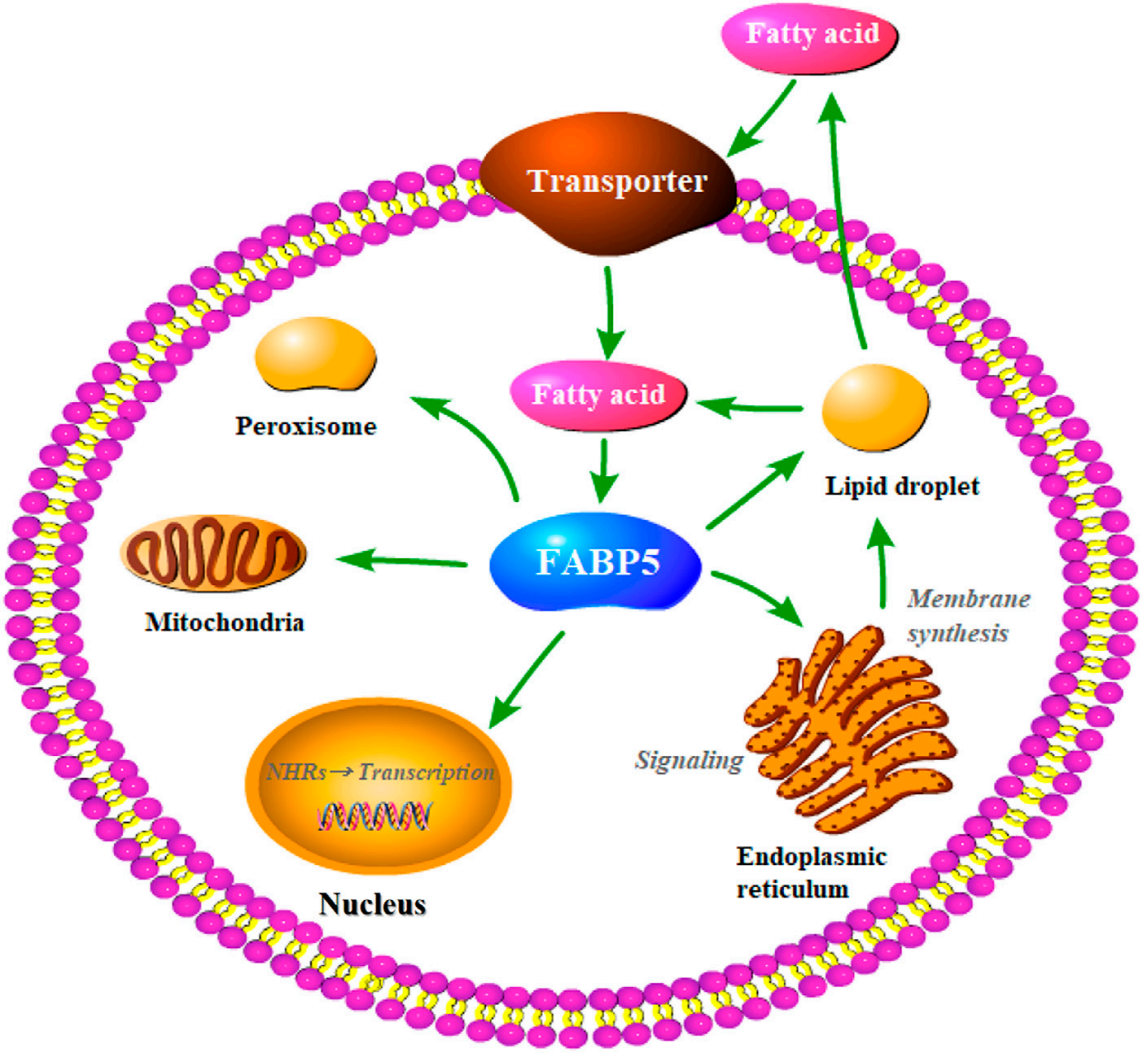
Putative functions of FABP5 in the cell. As lipid chaperones, FABP5 may actively facilitate lipid transportion to specific intracellular compartments and thereby realize different biological functions, such as the lipid droplet storage, signaling transduction, trafficking and membrane synthesis in endoplasmic reticulum, oxidation in mitochondria or peroxisome, lipid-mediated transcriptional regulation in the nucleus, or even signaling in an autocrine or paracrine manner outside the cell.

Fatty acid binding proteins (FABPs) are abundant cytoplasmic proteins (about 15 kD) expressed in most mammalian tissues and have specialized functions. The FABPs were originally known as intracellular proteins to buffer lipid (Chmurzynska, 2006). Previous studies have reported that they play important roles in the transportation and metabolism of fatty acids, and are strongly associated with metabolism disorders and abnormal cell proliferation under pathological state (Maeda et al., 2005; Iso et al., 2013). Fatty acid binding protein 5 (FABP5) which belongs to the FABPs family has been the subject of numerous recent studies. As a lipid chaperone, FABP5 may actively facilitate the transportation of lipids to specific intracellular compartments. This helps in the activity of several biological functions such as signal transduction, lipid droplet storage, trafficking and membrane synthesis in the endoplasmic reticulum, oxidation in the mitochondria or peroxisome, regulating the activity of cytosolic and other enzymes, and lipid-mediated transcriptional regulation in the nucleus (Figure 1). In addition, it is also involved in autocrine or paracrine signaling outside the cell, thereby enhancing the effects on systematic glucose, lipid homeostasis, energy metabolism, cell proliferation, and the immunological system. Several studies have reported that FABP5 plays a pivotal role in various diseases including metabolism disorders (e.g., obesity, insulin resistance, and T2DM) (Yeung et al., 2008), skin diseases (e.g., psoriasis) (Dallaglio et al., 2013), neurological diseases (e.g., Alzheimer’s disease) (Low et al., 2020), and carcinoma (e.g., prostate cancer, breast cancer, and cervical cancer) (Guaita-Esteruelas et al., 2018), due to its aberrant expression under pathological conditions. This indicates that FABP5 has tremendous potential in clinical applications.

This review has systematically summarized the most recent studies which have reported the important roles and corresponding mechanisms of FABP5 in various biological processes including lipid metabolism, lipid homeostasis, cell growth and differentiation, immune response, tumorigenesis, and cancer progression, skin, and neurological diseases. In addition, the possibility of applying FABP5 as a new diagnostic biomarker and therapeutic target in several diseases has been discussed.

## Biological Properties of FABP5

### The Structure and Functions of FABPs Family

FABPs are small secreted proteins that are widely found in most mammalian cells (Chmurzynska, 2006). Previous studies have reported that the whole FABPs family has nine isoforms including L-FABP/FABP1 (liver), I-FABP/FABP2 (intestinal), H-FABP/FABP3 (heart), A-FABP/FABP4/aP2 (adipocyte), E-FABP/FABP5/mal1 (epidermal), IL-FABP/FABP6 (ileal), B-FABP/FABP7 (brain), M-FABP/FABP8 (myelin), and T-FABP/FABP9 (testis) **(** Table 1). The isoforms are grouped according to the organs or tissues in which they were initially identified, isolated, and where they are most predominant. In other words, most tissues express multiple FABP isoforms and the expression of FABPs has no tissue specificity, which resulted in confusion and misunderstanding (Furuhashi and Hotamisligil, 2008).

**TABLE 1:** The features of FABP family members.

Gene	Common name	Aliases	Cytogenetic location	Number of amino acids	HGNC ID/OMIM ID	Molecular mass (Da)
FABP1	Liver FABP	L-FABP	2p11.2	127	3555/134650	14208
FABP2	Intestinal FABP	I-FABP	4q26	132	3556/134640	15207
FABP3	Heart FABP	H-FABP, O-FABP	1p35.2	133	3557/134651	14858
FABP4	Adipocyte FABP	A-FABP, aP2	8q21.13	132	3559/600434	14719
FABP5	Epidermal FABP	E-FABP, psoriasis-associated-FABP (PA-FABP), keratinocyte-type FABP (KFABP)	8q21.13	135	3560/605168	15164
FABP6	Ileal FABP	I-15P, Ileal lipid-binding protein (ILLBP), intestinal bile acid-binding protein (I-BABP)	5q33.3	128	3561/600422	14371
FABP7	Brain FABP	B-FABP, brain lipid-binding protein (BLBP)	6q22.31	132	3562/602965	14889
FABP8	Myelin FABP	MP2, peripheral myelin protein 2 (PMP2), M-FABP	8q21.13	132	9117/170715	14909
FABP9	Testis FABP	PERF, T-FABP, PERF15, testis lipid-binding protein (TLBP)	8q21.13	132	3563/-	15093

**FIGURE 2:**
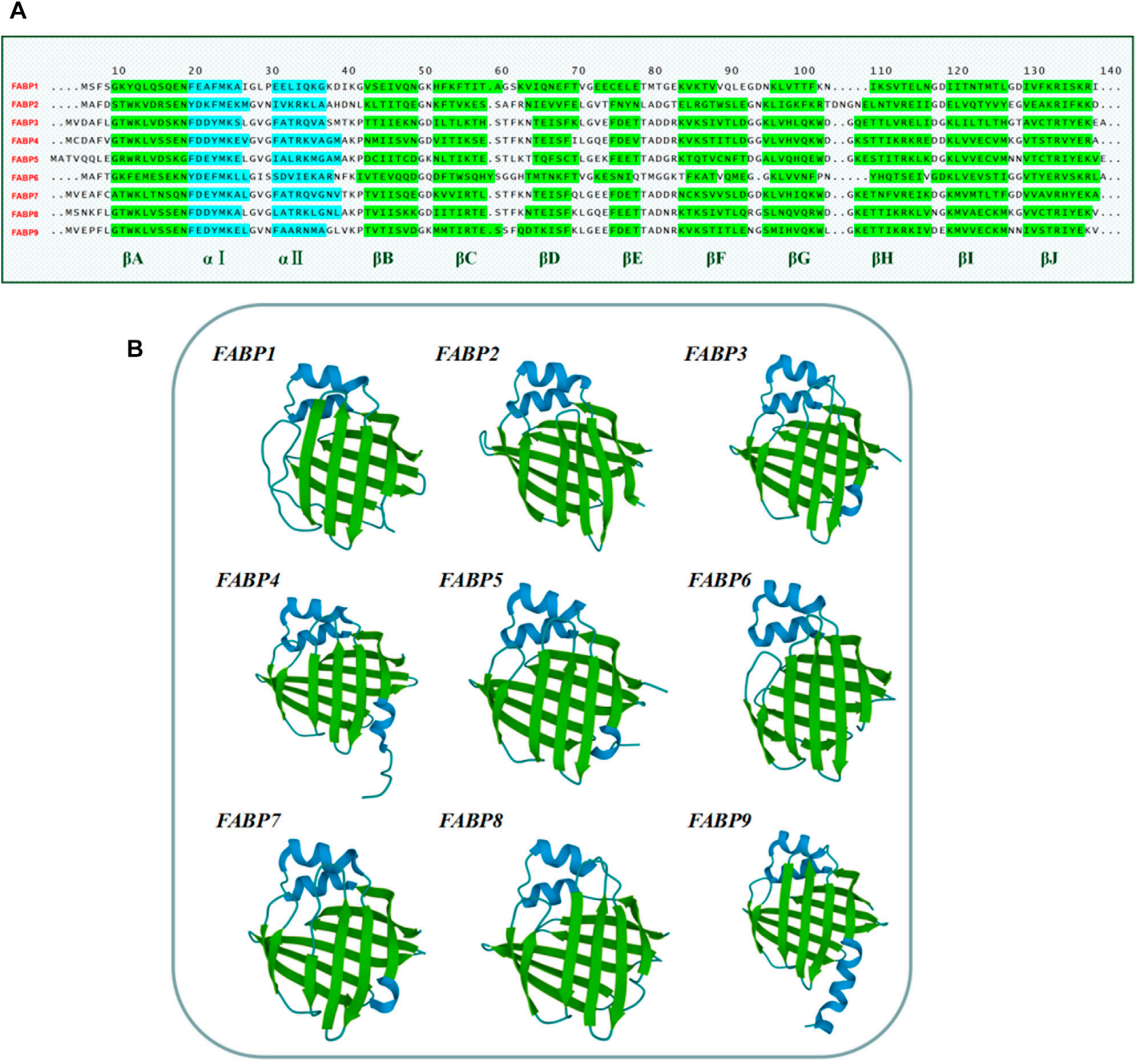
Amino acid sequences and tertiary structure of the human FABP family members. **(A)** Amino acid sequences of the human FABPs. Amino acid sequences were obtained from the National Center for Biology Information (NCBI) website (www.ncbi.nlm.nih.gov/). **(B)** The tertiary structure of the human FABP family members 1–9. The structures were adjusted to a similar angle. The structures were obtained from RCSB PDB (Research Collaboration for Structural Bioinformatics, Protein Data Bank) (https://www.rcsb.org/structure/). FABP1 (PDB code: 1LFO), FABP2 (PDB code: 3IFB), FABP3 (PDB code: 4TKB), FABP4 (PDB code: 3P6D), FABP5 (PDB code: 4AZQ), FABP6 (PDB code: 5L8I), FABP7 (PDB code: 1FDQ), FABP8 (PDB code: 1PMP), FABP9 (PDB code: 4A60).

The structure of the FABP family also contains vitamin A derivative-specific binding proteins including cytosolic retinol-binding protein (CRBPI/CRBPII/CRBPIII/CRBPIV) and cytosolic retinoic acid-binding protein (CRABPI/CRABPII) (Noy, 2000). The entire FABP family members share a highly conserved set of gene structures consisting of four exons separated by three introns. There is a similar position of exons and introns while the length of the introns between different subtypes is varied (Zimmerman and Veerkamp, 2002). Several studies have reported that the tertiary structures of FABP proteins are virtually superimposable despite the members exhibiting 22–73% amino acid sequence similarity (Banaszak et al., 1994; Storch and Thumser, 2010) (Figure 2A). The tertiary structure of FABPs comprises two α-helices as well as ten anti-parallel β-strands (Figure 2B). The available studies hypothesize the portal for fatty acids (FA) access and egress as a dynamic region made of α-helix II and the turns between βC-βD and βE-βF loops. The helix-turn-helix/portal region of FABP is considered a critical region that determines numerous functions of this protein family (Storch and Mcdermott, 2009).

**FIGURE 3:**
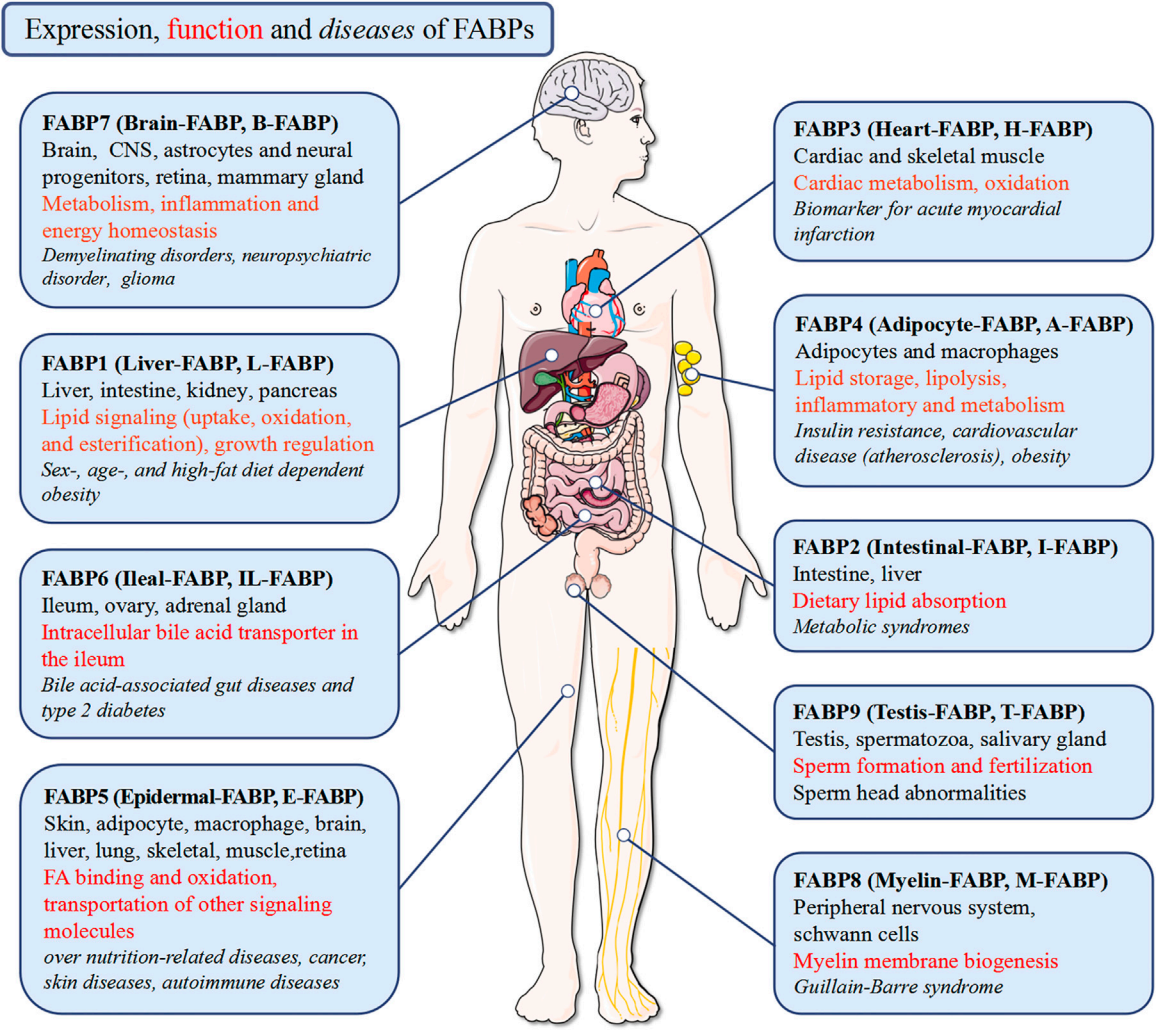
Expression, function and diseases of FABPs. FABPs function pleiotropically in human body to maintain tissue homeostasis in health and to participate in disease pathogenesis.

Dictated by the characteristic structure, the main function of the FABPs is to bind fatty acids, as well as the intake, transportation and consumption, despite their different selectivity, affinity, and binding mechanism (Storch and Thumser, 2000; Smathers and Petersen, 2011; Zhao et al., 2017). Structure-function studies of AFABP and KFABP reveal these specific ligands binding to FABP result in subtle conformational changes on its portal surface, promoting specific interactions and ultimately determining the functions of each FABP (Gillilan et al., 2007; Storch and Mcdermott, 2009). Consequently, the FABPs, including FABP3 (Zhuang et al., 2019), FABP4 (Furuhashi, 2019), FABP5 (Sharifi et al., 2013), FABP6 (Duggavathi et al., 2015), FABP7 (Sharifi et al., 2013; Ebrahimi et al., 2016; Xie et al., 2020), FABP9 (Moradi et al., 2019), play a systemic effect on the body’s lipid and energy metabolism by regulating FA transport in the nuclear and extra-nuclear compartments of the cell (Atshaves et al., 2010; Li et al., 2020) (Figure 3).

### Distribution and Features of FABP5

FABP5 is also known as epidermal FABP (E-FABP), cutaneous fatty acid-binding protein (C-FABP), psoriasis-associated FABP (PA-FABP), keratinocyte FABP (K-FABP), intracellular lipid-binding protein (iLBP), and mal-1. FABP5 was first discovered in the keratinocytes of psoriatic lesions, followed by characterization in the epidermis (Madsen et al., 1992). It is located at chromosome (Chr) 8q21. Immunohistochemistry results obtained from previous studies have indicated that FABP5 is expressed in a plethora of tissues or organs including the skin, eyes, lungs, spleens, kidneys, brain, liver, bone, tongue, adipose tissue, breast, retina tissue, cardiac, and intestinal tissue (Masouyé et al., 1997; Smathers and Petersen, 2011).

**FIGURE 4:**
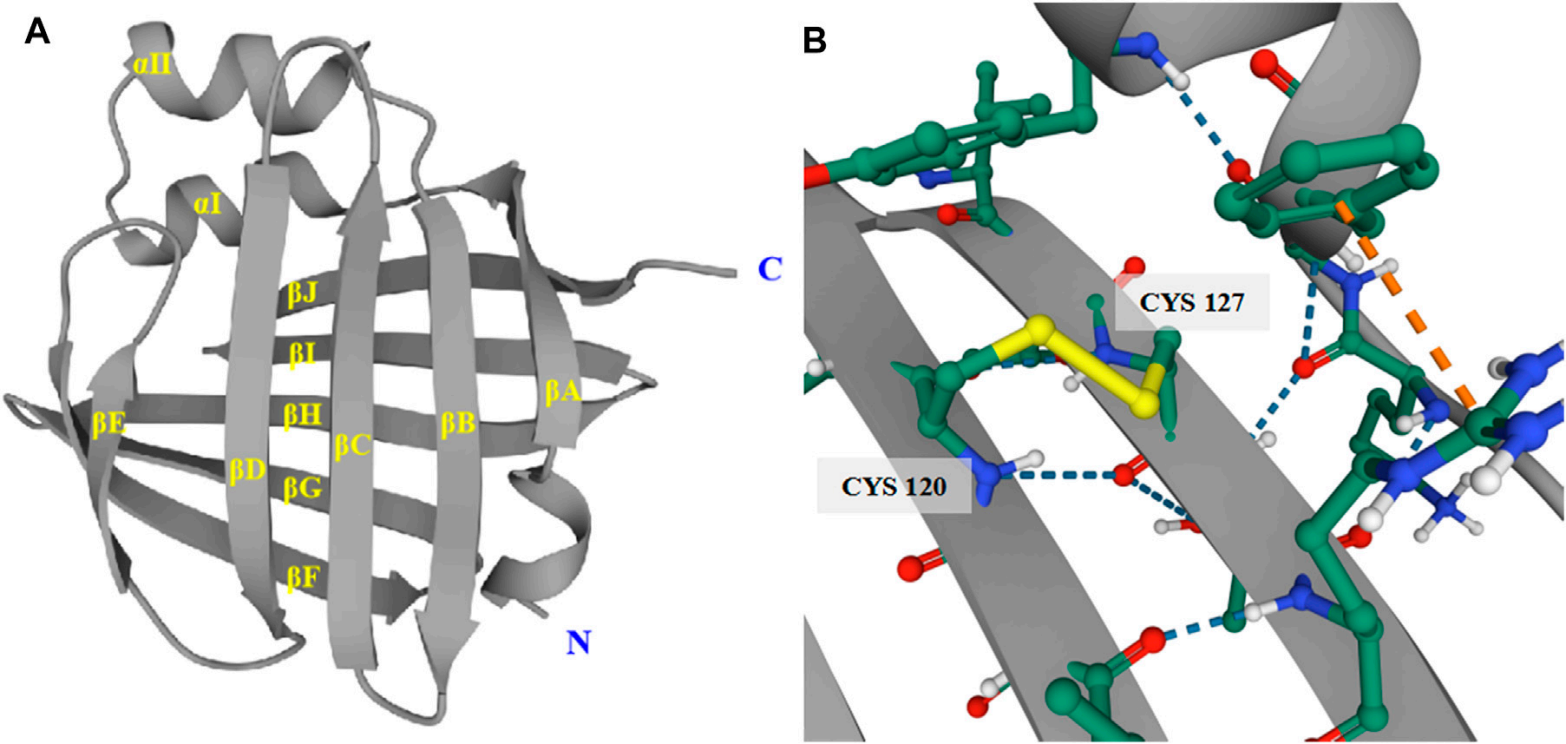
Tertiary structure of human FABP5. **(A)** The tertiary structure of FABP5 consists of 2 α-helices and 10 anti-parallel β-strands. **(B)** The disulphide bridge between cysteines 120 and 127 in FABP5 promotes protein stability.

The major function of FABP5 is to regulate the intracellular levels of fatty acids and specific metabolic pathways. However, FABP5 has several extra features. One remarkable difference is that the electrophoretic mobility of recombinant human FABP5 is different from that of FABPs and CRBPs within the scope of pI = 6.2–6.4 (Hohoff et al., 1999). In addition, there are six cysteines and one disulphide bridge between cysteines 120 and 127 in FABP5, which promote protein stability (Figure 4). Several studies have reported that the disulphide bridge may be associated with physiological and pathophysiological functions such as relieving oxidative damage to the epidermis and other tissues through the thiol-disulphide exchange reaction (Hohoff et al., 1999; Odani et al., 2000). Human FABP5 is highly polymorphic. With the exception of the general binding and trafficking functions, FABP5 is also diverse and hyperspecific in modulating the metabolism and function of its binding ligands. However, the existence of extra FABPs in several tissues makes it hard to define the exact function of FABP5, as well as its physiological ligand and underlying mechanism, which requires further research.

## Biological Functions of FABP5

### The Role and Mechanism of FABP5 in Lipid Metabolism

#### Fatty Acids Uptake and Transport

Fatty acids are the main building blocks of several lipid species including sphingolipids, phospholipids, and triglycerides. They can synthesize more complex lipid species through various metabolic pathways, which conduce to the numerous structural diversity of the cellular lipid pool. Studies have reported that the intracellular trafficking of fatty acids is a complicated and dynamic process that directly or indirectly influences multiple functions of the cell and especially regulates important biochemical processes in normal cells (Koundouros and Poulogiannis, 2020), including gene expression modulation, cell development, metabolism, and inflammatory response through enzymatic and transcriptional networks (Saltiel and Kahn, 2001; Hotamisligil, 2006). However, due to the comparative insolubility of these fatty acids and their underlying toxicity in free forms, FABPs are pivotal as buffer lipids (Savary et al., 2012; De Carvalho and Caramujo, 2018).

**FIGURE 5:**
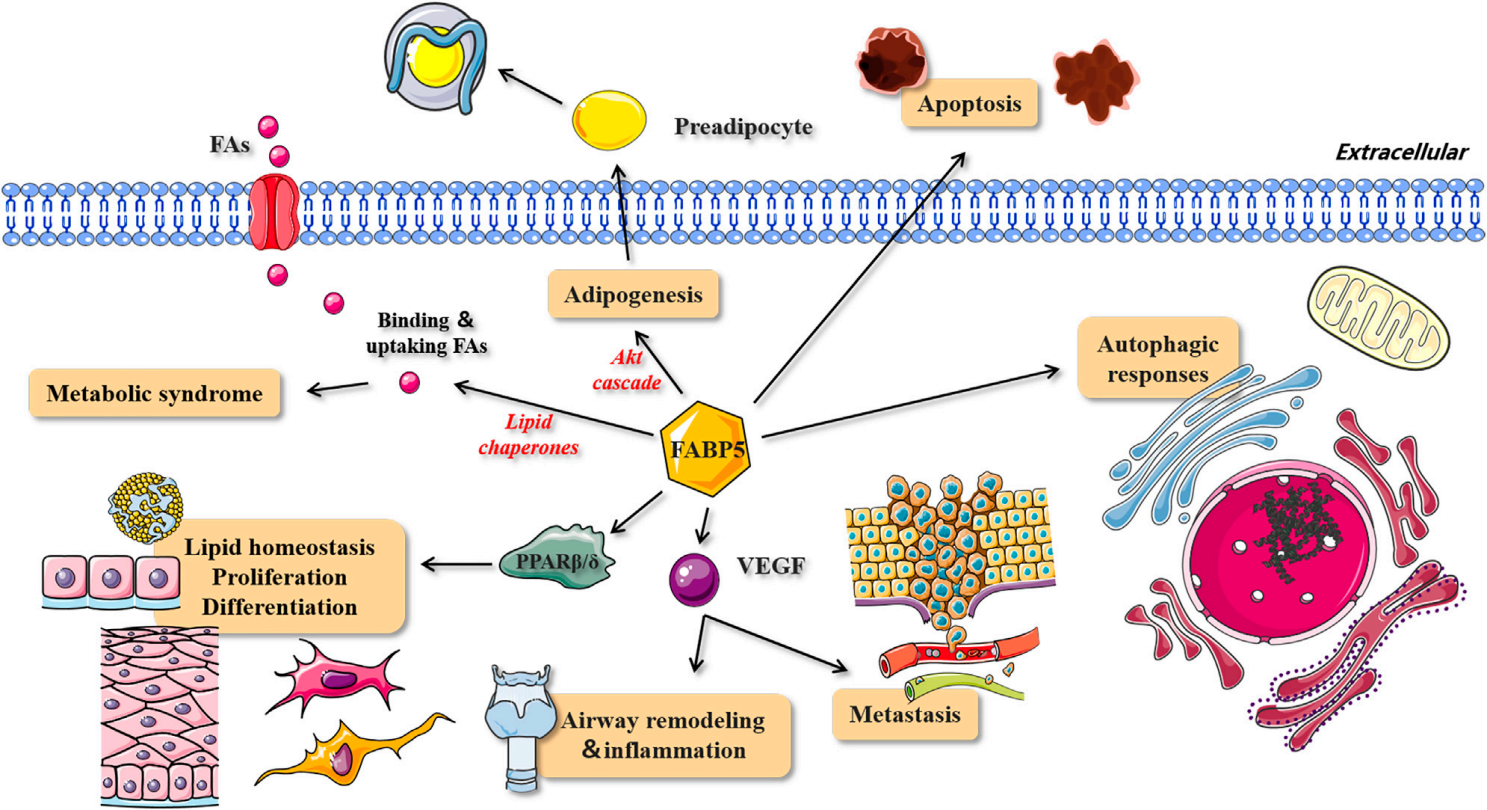
The role and mechanism of FABP5 in lipid metabolism, homeostasis, cell proliferation and differentiation. The roles and corresponding signal pathways of FABP5 in metabolic syndrome, adipogenesis, lipid homeostasis, proliferation, differentiation, metastasis, airway remodeling and inflammation, apoptosis and autophagic responses. FAs, fatty acids; VEGF, vascular endothelial growth factor.

As a lipid chaperone (Figure 5), FABP5 is considered as an important media of intracellular fatty acids, and can increase the solubility of fatty acids and reversibly combines with high-affinity hydrophobic ligands such as saturated and unsaturated long-chain fatty acids (LCFAs) (Coe and Bernlohr, 1998; Zimmerman and Veerkamp, 2002). It also actively facilitates the transport of fatty acids to specific compartments of the cell, for instance, from the cytoplasm to organelles (Iso et al., 2013). In addition, FABP5 interacts indirectly with membranes, enzymes, ion channels, receptors, or genes by modulating the concentrations of fatty acids, their CoA and carnitine esters, and other lipid mediators, thereby influencing various cellular processes (Glatz and van der Vusse, 1996). Iso et al. (2013) reported that capillary endothelial FABP4 and FABP5 participate in the uptake of circulating FAs into cardiac and skeletal myocytes to maintain sufficient ATP production (Iso et al., 2013). The study also demonstrated that FABP5 contributes to the formation of dipalmitoyl phosphatidyl choline (DPPC), the main surfactant phospholipid which is produced and secreted by lung type II alveolar cells (Guthmann et al., 2004), due to its high affinity for palmitate (Guthmann et al., 1998). Knockdown of FABP5 mRNA leads to the following significant alterations in human retinal pigment epithelial cells (ARPE-19): 1) suppression of fatty acids uptake and deceleration of FFAs metabolism, 2) alteration of cellular lipid composition and an increase in cellular lipid droplets, and 3) decreasing level of apolipoprotein B100, which is synthesized and secreted by ARPE-19 cells and may serve as a backbone structure for the formation of lipoprotein particles in these cells (Wu et al., 2010). These findings suggest that FABP5 operates as a key fatty acids transporter and plays a critical role in lipid metabolism.

### Lipid Metabolism and the Occurrence of Metabolic Syndrome

Metabolic syndromes such as obesity, high uric acid, high blood fat, hypertension, type II diabetes, and atherosclerosis, have received increasing attention due to the great changes that have occurred in eating habits and the general lifestyle (Figure 5). Accumulating evidence shows that the level of FABP5 may be closely associated with the pathogenesis of chronic metabolic diseases through its expression in adipocytes and macrophages (Hotamisligil et al., 1996; Maeda et al., 2005; Cao et al., 2006).

Yeung et al., (2008) reported that the serum FABP5 level is positively associated with the parameters of body fat contents (BMI, hip, and waist circumference), poor blood lipid status (high triglycerides and low HDL-C), and hyperinsulinemia. The same correlation was reported in other previous studies (Yeung et al., 2008; Zhang et al., 2020). Notably, a statistical analysis of 225 men reported that FABP5 is an important independent risk factor for carotid intima-media thickness, which indicates that FABP5 is relevant to cardiovascular metabolic risks. In addition, atherosclerotic low-density lipoprotein (LDL) receptor-deficient mice treated with a Western-style high-cholesterol diet enhanced the expression of FABP5, as well as plasma levels of atherogenic lipoproteins, very low-density lipoprotein (VLDL), and LDL (Hoekstra et al., 2006). Similarly, when fed on a Western-style diet, survival of FABP4^–/–^FABP5^–/–^ApoE^–/–^mice (FABP4 and FABP5 co-deficient mice crossed into ApoE^−/−^ model) were found to significantly increase, which may be due to increased plaque stability (Boord et al., 2004; Ibarretxe et al., 2016). Furthermore, the low-density action of circulating FABP5 led to a decreased cholesterol efflux capacity (CEC) from macrophages, which is the first step in the reverse cholesterol transport pathway and carotid atherosclerosis. This suggests that the serum FABP5 concentration is a regulatory factor of CEC and a potential biomarker for residual risk of atherosclerosis (Furuhashi et al., 2017). These results suggest a particular role of FABP5 in atherogenesis. Bagheri et al. (2010) reported, for the first time, the link between FABP5 and metabolic and inflammatory cardiovascular disease (CVD) risk factors in humans. The study pointed out that FABP4 and FABP5 synergistically promote inflammatory, metabolic, and atherogenic processes, suggesting their roles in representing mediators and biomarkers of metabolic and CVD disease in T2DM (Bagheri et al., 2010). Further studies reported that a combined deficiency of FABP4 and FABP5 does not reduce weight gain or adiposity but it improves glucose tolerance and insulin sensitivity, and may prevent the onset of fatty liver. Therefore, although limited under some conditions, FABP5 shows potential importance in controlling systematic metabolism (Cao et al., 2006; Hotamisligil and Bernlohr, 2015).

Analysis of FABP5^−/−^mice indicated the necessity of FABP5 for enteroendocrine cell development due to the finding which indicated that genetic depletion of FABP5 is linked with a significant reduction in plasma glucose-dependent insulinotropic polypeptide (GIP) concentration, which probably stems from impaired neural regulation of GIP secretion (Yamane et al., 2016). GIP, also known as a gastric inhibitory polypeptide, is produced by a subset of enteroendocrine cells known as K cells. GIP is a hormone that induces the postprandial insulin response and affects nutrient intake, especially fat (Shibue et al., 2015). This remarkable observation reconfirmed the contribution of FABP5 in the lipid metabolism and development of obesity. The finding also provided strong support for FABP5’s function in the prevention and treatment of metabolic diseases.

Interestingly, Hertzel et al. (2002) reported contradictory results. There were no significant differences in overall morphology, weight, and serum-free fatty acid concentrations between wild-type mice and FABP5 transgenic mice. In addition, both basal and hormone-stimulated lipolysis were increased in adipocytes of FABP5 transgenic mice (Hertzel et al., 2002). These results were consistent with the results obtained in another study done by Hertzel et al. (2006) which reported that overexpression of FABP5 in adipose tissues causes an increase in lipolysis and a decrease in cellular free fatty acid levels (Hertzel et al., 2006). Triglycerides must be mobilized by lipolysis before re-esterification until they can be reassembled on the endoplasmic reticulum and thus incorporated into lipoprotein particles (Gilham et al., 2005). Therefore, the presence of FABP5 may cause an increment in the efficacy of fatty acid release and promote the re-esterification of liberated fatty acids in the endoplasmic reticulum. This may be a key step explaining why the overexpression of FABP5 results in increased lipolysis. Furthermore, it might also reasonably explain why FFAs and triglycerides are increased in ARPE-19 cells by 18% and 67%, respectively, after siRNA treatment (Wu et al., 2010).

The mechanism underlying the close correlation between insulin resistance and lipid metabolism is getting clearer (Rachek, 2014; Athyros et al., 2018). Fatty acids can stimulate insulin secretion when transiently elevated, and can lead to pancreatic islet failure, lipotoxicity, and apoptosis when chronically elevated (Poitout et al., 2006; Acosta-Montaño et al., 2019). Studies conducted on FABP4 null mice with induced obesity suggested that the metabolism of adipocyte fatty acids is a crucial component of the mechanisms leading to systemic insulin resistance in obesity (Hotamisligil et al., 1996; Shaughnessy et al., 2000). Both FABP3 and FABP5 are thought to be associated with the stimulus-secretion coupling mechanisms and insulin secretion process (Hyder et al., 2010). The adipocytes of FABP5-deficient mice exhibited an enhanced capacity for the transport of insulin-dependent glucose and a modest increase in systemic insulin sensitivity while overexpressing FABP5 aggravated insulin resistance and hyperglycaemia (Maeda et al., 2003). Further profiling of the lipids indicated an increase in the number of shorter-chain (C14) fatty acids and a decrease in the number of longer-chain (C18 or C20) fatty acids in the adipose and muscle tissues of FABP4^–/–^FABP5^–/–^ mice, which favors the enhancement of insulin receptor signaling and insulin-stimulated glucose uptake (Maeda et al., 2005). Experiments conducted on the structure-binding relationship have also found that FABP5 has a high affinity for stearic acid, which decreases when the number of carbon atoms decreases or when double bonds are introduced into the fatty acid chain (Siegenthaler et al., 1994). These observations probably suggest that the distribution and availability of intracellular fatty acids and their derivatives may be more critical than the absolute amounts of fatty acids.

In addition to the vital role of FABP5 in binding FFAs and moderating lipid metabolism and transport, it is also closely associated with adipogenesis. Specifically, FABP5 maintains the viability of pre-adipocytes by activating the Akt cascade, and reduced FABP5 expression induces apoptosis of differentiated pre-adipocytes through caspase-3 activation (Ma X. et al., 2010) (Figure 5).

### The Role and Mechanism of FABP5 in Lipid Homeostasis

#### Interaction With PPAR Nuclear Receptors and Utilization of Retinoic Acid

Fatty acid nuclear receptors, known as peroxisome proliferator-activated receptors (PPARs), are also regarded as members of the nuclear hormone receptor super-transcription factor family, including three isotypes (PPARα, PPARβ/δ, and PPARγ). Their main function is to modulate lipid homeostasis and have a profound impact on cellular proliferation and differentiation during tumorigenesis (Schoonjans et al., 1996) (Figure 5). Accumulating evidence has confirmed the direct physical interactions between FABP5 and PPARs (Samulin et al., 2008). FABP5 is controlled by these transcription factors. Tan et al. (2002) reported that FABP5 selectively enhances the activities of PPARβ/δ and this cell signaling practically impacts the differentiation of keratinocytes. This finding suggested potential functional cooperation between FABP5 and PPARs in regulating the transcriptional activities of their mutual ligands (Tan et al., 2002). As a small intracellular protein, FABP5 massively relocates to the nucleus and potentially targets fatty acids to transcription factors, PPARβ/δ, and PPARγ in the nuclear lumen (Tan et al., 2002), thereby allowing PPARs to perform their biological functions. This is one mechanism of how fatty acids act as signaling molecules for conveying messages to the nucleus after being taken up by the cell (Wolfrum et al., 2001). However, the exact mechanism for the translocation of FABP5 to the nucleus upon ligand binding and the specific process of PPAR activation by FABP5 in the nucleus has not yet been elucidated. One study hypothesized that the fatty acid-activated nuclear receptor PPAR links intracellular fatty acid levels to gene expression by binding to its response element (PPRE) in the promoter of a target gene (Tan et al., 2002).

Interestingly, peritoneal macrophages isolated from FABP5-deficient mice exhibited a clear increase in PPARγ activity. FABP5 deficient T cells also exhibited an elevated PPARγ expression. Moreover, FABP5 was found to play a pro-atherogenic role by suppressing PPARγ activity (Babaev et al., 2011). A later study suggested the dual effects of FABP5 on PPARγ activity, that is, FABP5 functions as a positive regulator of PPARγ under normal culture conditions, but inhibits the activity of PPARγ in serum deprivation (Yu et al., 2016).

Retinoic acid (RA) is known to be a ligand for PPARβ/δ and the classical RA receptor (RAR), which is necessary for the regulation of critical biological processes including differentiation, apoptosis, and cell survival (Pavone et al., 2011). RA has been shown to induce gene expression that affects lipid and glucose homeostasis by activating PPARβ/δ, thereby enhancing lipid oxidation, energy dissipation, and insulin responses (Noy, 2016). Obesity leads to the down-regulation of adipose PPARβ/δ expression *in vivo*. One study reported that the implantation of RA into obese mice resulted in up-regulated PPARβ/δ levels followed by subsequent weight loss and elevated expression of the insulin-signaling gene PDK1 (Wolf, 2010). Therefore, RA is potentially effective in preventing diet-induced obesity and insulin resistance using two distinct mechanisms: counteracting adipogenesis and promoting energy expenditure (Noy, 2013).

FABP5 conveys RA from the cytosol into the nucleus to bind to PPARβ/δ, and the cellular retinoic acid-binding protein II (CRABPII) carries RA into the nucleus to bind to RAR (Wolf, 2008). It is worth noting that RA activates RAR in cells that express a low FABP5/CRABPII ratio, otherwise it targets PPARβ/δ (Levi et al., 2015). Recent studies in triple-negative breast cancer (TNBC) have proved that decreasing the proportion of FABP5/CRABPII in breast tissue converts RA from PPARβ/δ to RAR, thereby suppressing tumor growth. Therefore, the proportion of FABP5/CRABPII determines whether RA inhibits cell proliferation *via* the CRABPII/RAR signaling or facilitates tumor progression *via* the FABP5/PPARδ pathway (Liu et al., 2011) (Figure 5).

### Up-Regulating the Expression of VEGF and Dual Role on Endothelial Cell Fate

Vascular endothelial growth factor (VEGF) is one of the most effective stimulating factors for angiogenesis and is thus involved in the progression of angiogenesis-related diseases, such as facilitating the malignant dissemination of the primary tumor cells (Neufeld et al., 1999). VEGF regulates most of the endothelial responses, especially endothelial cell proliferation and migration (Matsumoto and Ema, 2014) (Figure 5).

The regulation of endothelial cell homeostasis and vascular integrity is necessary for normal organ functions such as tissue repair and regeneration (Huang et al., 2019). A study demonstrated the presence of enriched FABP5 expression in aging human dermal microvascular endothelial cells obtained from elderly skin tissues (Ha et al., 2004). It also revealed the expression of FABP5 in the endothelial cells of some larger vessels, involving arteries and veins. This finding could be a valuable supplement to earlier findings that endothelial cell-FABP5 expression is confined to the microvasculature of some tissues in the kidney, heart, and placenta (Masouyé et al., 1997). A large amount of FABP5 expression has also been observed in endothelial cells within cutaneous hemangiomas (the most frequent endothelial cell-derived tumors). FABP5 also promotes the chemotactic migration and angiogenic sprouting of endothelial cells both *in vitro* and *ex vivo* (Yu et al., 2016). However, the FABP5 level shows no response to VEGF, despite studies proving that the FABP5 expressed in endothelial cells and VEGF significantly enhances endothelial cell proliferation. This indicates that FABP5 is not a downstream target of VEGF, instead, it is probably regulated by the extracellular lipids in endothelial cells. In addition, FABP5 may promote apoptosis resulting in vascular regression when meeting the conditions of nutrient deprivation, such as tissue ischemia or starvation, or induced by other stressors (Figure 5). A recent study suggested that autophagic response may be a potential explanation, however, the hypothesis needs to be addressed in future studies (Yu et al., 2016) (Figure 5). Interestingly, opposite results were obtained in other studies demonstrating that FABP5 induces metastasis of rat mammary epithelial cells (Rama 37 cells) through the up-regulation of VEGF expression (Jing et al., 2001) (Figure 5). In line with this observation, another study confirmed that the expression of FABP5 is involved in airway remodeling and inflammation in asthma by inducing VEGF production, and a positive correlation between FABP5 and VEGF levels was also observed in those circumstances (Suojalehto et al., 2015) (Figure 5).

### The Role and Mechanism of FABP5 in Cell Growth and Differentiation

#### FABP5 Promotes Cell Proliferation and Differentiation

FABP5 was reported to regulate the developmental gene expression programs induced by lipid messengers such as RA and efficiently promotes cell proliferation by activating PPARδ (Yu et al., 2012; Levi et al., 2013). Accumulating evidence has shown that FABP5 is closely correlated with cellular proliferation and differentiation (Figure 5). Yu et al. (2016) reported that FABP5-knockdown (FABP5-KD) in epithelial cells had a profound impairment of cell proliferation. Further cell cycle analysis revealed that the percentage of FABP5-KD cells was significantly increased in the G0/G1 phase with subsequent decreases in the S and G2/M phases when compared with control cells. This indicates that epithelial cell-FABP5 can be envisioned as a potent positive regulator of cell cycle progression under normal circumstances (Yu et al., 2016).

Moreover, FABP5 has been shown to regulate melanocyte proliferation and keratinocyte differentiation (Siegenthaler et al., 1994). FABP5 is also involved in the differentiation of several cell types such as T helper cells and neural cells. In addition, FABP5 stimulates the differentiation of PC12 cells by promoting neurite extension in the neural system (Liu et al., 2008) and regulating the growth and/or death of septoplasty in the growth plate cartilage of mice (Bando et al., 2017).

### The Role of FABP5 in Neurite Outgrowth and Axon Development

Considering the role of FABP5 in neurite outgrowth, Liu et al. (2015) found that cells lacking FABP5 are less responsive to nerve growth factor (NGF) treatment than control cells as manifested by a significant reduction of neurite expression. The study also reported that FABP5 is up-regulated by NGF and is vital for NGF-induced and polyunsaturated fatty acid-potentiated neurite outgrowth (Liu et al., 2015). A further study reported that FABP5 is expressed in retinal ganglion cells (RGCs) since these cells reached the ganglion cell layer, thereby unraveling the functional role of FABP5 in the elaboration of RGC axons in both development and regeneration (Allen et al., 2001).

In contrast to FABP3, the expression of FABP5 is dominant in the glia of the pre-natal and peri-natal brain (Owada et al., 1996). The differential temporal expression of FABP5 during the development can be attributed to its proposed role in transporting long chain-free fatty acids and/or other hydrophobic ligands during neuronal differentiation and axon growth (Liu et al., 2000). The differentiation of progenitor cells into mature neurons, the late stage of the RA-induced neuronal differentiation process, is mediated through PPARβ/δ (Yu et al., 2012) (Figure 5). Accumulating evidence has shown that FABP5 is also implicated in the neurogenesis, development of astrocytes, neuronal migration, and terminal differentiation of neurons, indicating that FABP5 is required for neuronal development in nervous tissues (Liu et al., 2000).

### The Role and Mechanism of FABP5 in Immune Response and Inflammatory Process

#### Expression of FABP5 in Immunocyte and Inflammatory Cells

**FIGURE 6:**
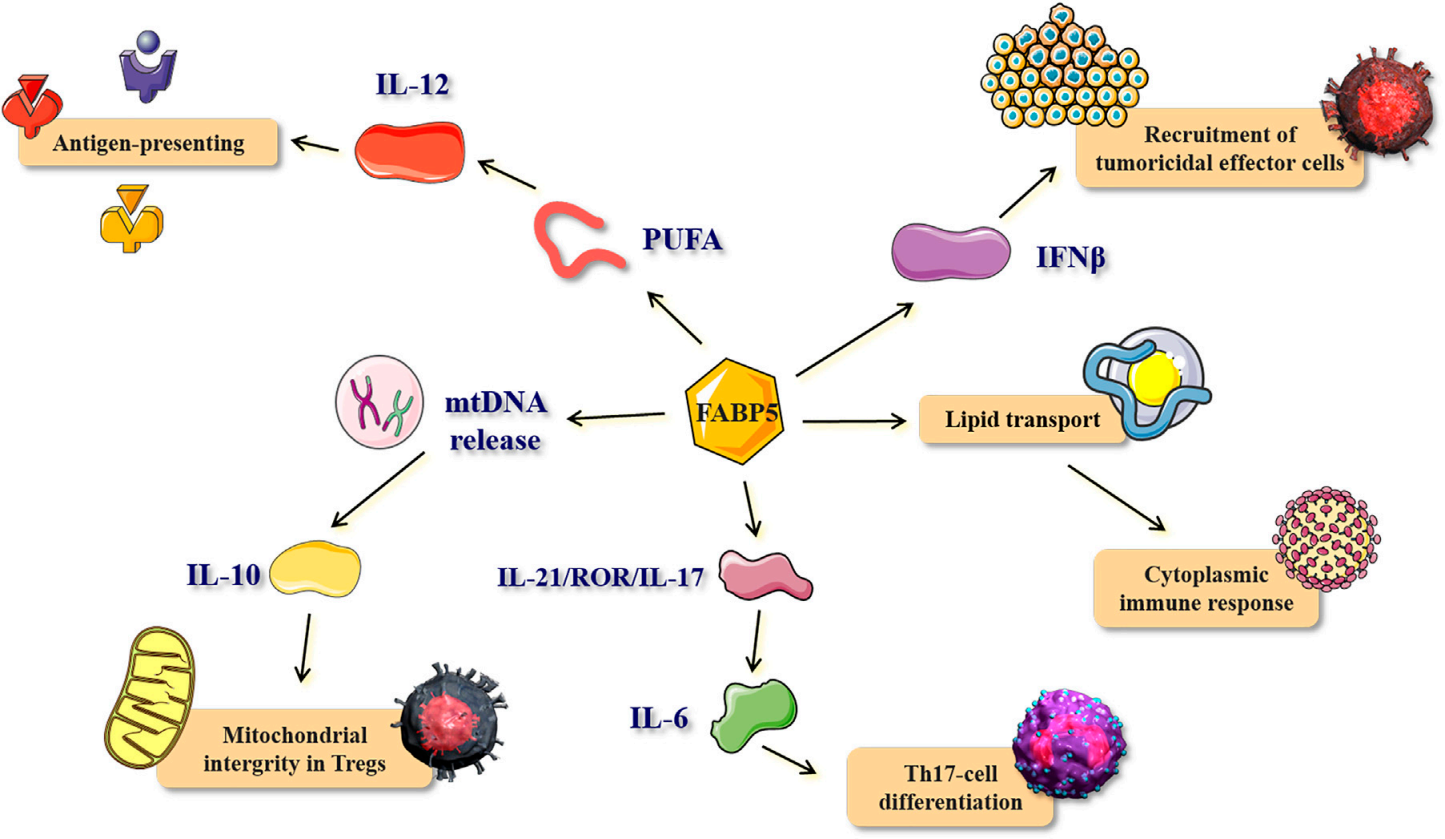
The role and mechanism of FABP5 in immune response and inflammatory process. The bio-function and corresponding mechanisms of FABP5 in antigen-presenting, recruitment of tumoricidal effector cells, cytoplasmic immune response, Th17-cell differentiation, and mitochondrial integrity in Tregs. PUFA, polyunsaturated fatty acids.

Several studies conducted in the last decade have provided evidence for the abundant expression of FABP5 in immune cells, especially in antigen-presenting cells (APC) and T cells (Reynolds et al., 2007; Li et al., 2009). Dendritic cells (DCs), the most potent APCs, have shown a special localization of FABP5 in the splenic white pulp *in vivo*. FABP5 expressed in DCs is involved in the process of IL-12 production through the modulation of intracellular polyunsaturated fatty acids (PUFA)-metabolism and/or -mediated signal transduction, thereby suggesting the possible involvement of FABP5 in the antigen-presenting function (Kitanaka et al., 2003; Kitanaka et al., 2006) (Figure 6). T-cell specific FABP5 modulates T-cell subset differentiation by influencing both transcriptional and metabolic programs that control T cell differentiation and effector functions.

FABP5 is also known to be highly expressed in macrophages. Tumor-associated macrophages (TAMs), derived from the circulating monocytes, are the primary component of the tumor microenvironment (TME), and they are associated with a poor prognosis since they promote tumor progression (Yin et al., 2019). A previous study reported that FABP5 is expressed in a specific subset of TAMs which exhibit the CD11b^+^F4/80^+^Ly6c^+^CD11c^+^MHCII^+^ phenotype. Furthermore, the recruitment of tumor-killing effector cells, especially NK cells, is enhanced by the expression of FABP5 in TAMs. FABP5 promotes the production and signaling pathway of interferon β (IFNβ), thereby achieving tumor killing (Rao et al., 2015b) (Figure 6). Mechanistically, FABP5 acts as a cytoplasmic energy carrier that may enhance the impacts of macrophage immune surveillance by boosting lipid droplet formation and IFN-β responses. This shows that FABP5 is essential in regulating host immune responses to tumor challenge and that host expression of FABP5 may represent a new anti-cancer protective factor by strengthening the antitumor activity of TAMs (Zhang et al., 2014). Similarly, another study reported that FABP5-mediated lipid transport encourages the immune cells to establish necessary lipid platforms (such as lipid droplets) that bind to specific signaling proteins to modulate the cytoplasmic immune response (Farese and Walther, 2009) (Figure 6). Interestingly, several studies have reported that the up-regulation of FABP5 promotes inflammatory autoimmune diseases such as experimental autoimmune encephalomyelitis (EAE) (Rao et al., 2015a) and Sjögren syndrome (SS) (Shinzawa et al., 2018), with their pathogenesis probably involving both Th1 and Th17 T helper cell subsets.

CD4^+^ and CD8^+^ T cells, also known as T helper (Th) and T cytotoxic (Tc) lymphocytes, respectively, are critical for the control of immune function mainly through releasing pro-inflammatory cytokines or exerting direct cytotoxic effects (Taniuchi, 2018). A previous study reported that the FABP5 in CD4^+^ T cells promotes Th17-cell differentiation, while counteracting the development of regulatory T cells (Tregs) (Li et al., 2009). This is vital for the control of IL-6 stimulation in the IL-21/ROR/IL-17 pathway which is regulated by FABP5 (E-FABP) (Figure 6). Field et al. (2020) further reported that FABP5 maintains mitochondrial integrity in Tregs. And the inhibition of FABP5 results in mtDNA release, which triggers expression of IL-10 and enhances the suppressive capacity of Tregs (Field et al., 2020) (Figure 6). Coincidentally, Park and Kupper, 2015) later reported that the expression of FABP4 and FABP5 in CD8^+^ tissue-resident memory T (TRM) cells, which indefinitely reside in epithelial barrier tissues and provide a rapid antigen-specific immune response upon reinfection, protects the host from the pathogens (Park and Kupper, 2015). The elevated expression of FABP4 and FABP5, and increased extracellular FFA uptake was also observed in human CD8^+^ TRM cells of normal and psoriatic skin, which was consistent with the data obtained from mice models. However, FABP4/FABP5-deficient skin CD8^+^TRM cells showed less protection against infection by a skin virus, and infection with the skin vaccinia virus (VACV) induced production of the lung FABP4^−/−^/FABP5^−/−^ CD8^+^TRM cells that are less effective in protecting mice from lethal lung challenge against VACV. All these results suggest that FABP5 plays a pivotal role in the maintenance, long-term survival, and protective function of CD8^+^ TRM cells (Pan et al., 2017).

In addition, FABP5 is found expressed in murine bone marrow-derived MCs (BMMCs), and plays a critical role in the production of TNF-α after lipopolysaccharide (LPS) treatment (Yamamoto et al., 2008). FABP5(+) cells were also reported as being widely distributed throughout the lymph node with well-developed lysosome and phagocytic materials contained in the cytoplasm (Tokuda et al., 2010), as well as cortical thymic epithelial cells (cTEC) which produce a variety of humoral factors such as cytokines and eicosanoids, thereby modulating thymocyte homeostasis and regulating the peripheral immune responses (Adachi et al., 2012).

### Roles of FABP5 in Immune Response and Inflammatory Process

FABP5 has an inseparable relationship with immune cell biology since lipid metabolism plays a key role in innate and adaptive immune responses and the profound effects of FABP5 on the pathways related to the signaling of lipid metabolism (Zhang and Li, 2014). Accumulating correlative evidence has stressed the important contribution of FABP5 in the regulation of immune function, which hassled to the underlying mechanisms becoming increasingly well understood (Westerbacka et al., 2007). A recent study investigated the response of lung epithelial cells to infection by the influenza virus, thereby adding more evidence on the link between FABP5 and the immune system. In the study, FABP5^−/−^ mice exhibited sustained tissue inflammation characterized by increased cell infiltration of macrophages and neutrophils when compared with wild-type mice, both infected with the influenza A virus. The adaptive immune response characterized by the accumulation of T and B cells in lung tissue and increased levels of H1N1-specific IgG antibodies was also increased in FABP5^−/−^ mice. These results support the speculation that FABP5, as a transporter of fatty acids, plays a crucial protective role against the oxidative damage to lipids during infection. Interestingly, the expression of FABP5 in wild-type lung tissue was decreased after infection with the influenza A virus, which is in conjunction with a decrease in the anti-inflammatory molecule PPAR-γ activity. A possible explanation for the biphasic expression of FABP5 is that the initial down-regulation is regarded as part of the immune response initiation, while the subsequent recovery of FABP5 expression levels is to aid attenuation of the inflammatory response (Gally et al., 2013b).

A recent study conducted on alveolar macrophages during acute rejection of rat lungs has for the first time provided evidence on the up-regulation of FABP5 expression in alveolar macrophages (AM) during severe inflammation (Holler et al., 2010). Monocytes, which accumulate in the graft vessels lumina of renal allografts, also express elevated FABP5 levels during rejection (Grau et al., 2003). All the evidence supports the idea that FABP5 is implicated in the immunological rejection response after allograft organ transplantation and thus it can be exploited to improve the outcome of transplantation.

However, another study reported that FABP5^−/−^ mice infected with *Listeria* monocytogenes showed no defect in clonal expansion, contraction, and formation of memory CD8^+^ T cells and CD4^+^ T cells when compared with wild-type mice. This indicates that FABP5 is not necessary for antigen-specific T cell responses after a bacterial infection (Li and Schmidt, 2016). Moreover, there is also a model in which cigarette smoking-induced FABP5 inhibition contributed to increased inflammation in COPD exacerbations, which is speculated to be as a result of decreasing PPARγ activity (Gally et al., 2013a; Rao et al., 2019).

Recent studies have reported that FABP5 limits the anti-inflammatory response, and the pharmacological inhibition of FABP5 reduce inflammation and pain (Abplanalp et al., 2020). An innovative study focusing on infected mice suffering acute inflammation reported a remarkable phenomenon where the inhibition of FABP5 reduced pain, edema, cytokine, and PGE2 levels, which is a major eicosanoid that increases pain in inflammatory settings (Bogdan et al., 2018) Endocannabinoid anandamide (AEA), also known as the endogenous lipid arachidonoyl ethanolamide, exerts neurobehavioral, cardiovascular, and immune-regulatory effects *via* cannabinoid receptors (CB) (Pacher et al., 2005). Elevated endocannabinoids levels can have beneficial pharmacological effects on stress, pain, and inflammation, as well as ameliorate the effects of drug withdrawal. Recent studies conducted in the last decade have elucidated the link between FABP5 and endocannabinoid anandamide (AEA) where FABP5 has been identified as an intracellular transporter of AEA (Sanson et al., 2014). Another study reported that FABP5 hydrolyzes AEA into arachidonic acid (AA) and ethanolamine, which is catalyzed by fatty acid amide hydrolase (FAAH), an enzyme localized in the endoplasmic reticulum (Kaczocha et al., 2009). The presence and overexpression of FABP5 significantly enhance pain and inflammation since AEA becomes inactivated after cellular uptake and subsequent catabolism, thereby losing the antinociceptive function. Therefore, the role of FABP5 in pain and inflammation positions FABP5 inhibitors as potential anti-inflammatory and analgesic drugs (Kaczocha et al., 2015).

### The Effect of FABP5 on Immune Factors and Inflammatory Cytokine

Blowout studies conducted in the past decade have shed light on the molecular function of immune factors and inflammatory cytokines (Gu et al., 2019; Sun et al., 2020). The studies have revealed that the pro-inflammatory response (M1) is to a large extent activated by interferon-γ (IFN-γ) and/or lipopolysaccharide (LPS), characterized by increased pro-inflammatory cytokines such as tumor necrosis factor α (TNF-α), interleukin 12 (IL-12), and IL-6, and decreased transforming growth factor β (TGF-β) (Ambarus et al., 2012). Alternatively, the non-classical anti-inflammatory (M2) response is originally triggered by IL-4, IL-13, and IL-10. Accumulating evidence suggests that FABP5 may be involved in the production of cytokines in macrophages and dendritic cells by controlling cellular lipid metabolism and signaling (Kitanaka et al., 2003). A previous study reported that FABP5 null macrophages display suppression of inflammatory genes, such as COX2 and IL-6 (Babaev et al., 2011). In addition, FABP5 null mice display higher mRNA levels of anti-inflammatory cytokines IL-10, arginase, YM1, and Fizz-1 in the liver when compared with wild-type mice. All these findings suggest that FABP5 functions as a major regulator of the pro-inflammatory response inextricably linked with its interactions with various distinct cytokines. It is also worth noting that the homeostasis of macrophage phenotypes (M1 and M2) is a key component in the inflammation process. Some researchers have hypothesized that deletion of FABP5 can produce higher levels of M2-related cytokines and genes, and the subsequent effect should be addressed in future studies (Moore et al., 2015).

### Relationship Between Lipid Metabolism and Inflammation Mediated by FABP5

Recent studies have reported that obesity is positively associated with a bacterial skin infection and inflammatory skin disorders including pruritus and seborrheic dermatitis. However, the underlying mechanisms have not yet been elucidated. FABP5 as a specific factor that is correlated with lipid metabolism and inflammation, presents a novel and viable idea for understanding obesity-associated inflammatory skin diseases in humans. FABP5 is up-regulated in macrophages and keratinocytes in skin tissues during a high-fat diet, thereby significantly instigating inflammatory skin lesions in the obesity mouse model. This suggests that FABP5 might represent a molecular sensor for triggering high fat diet-induced skin inflammation (Zhang et al., 2015). Furthermore, FABP5 deficiency abrogated high-saturated-fat diet-induced skin lesions in obese mouse models *in vivo* (Zeng et al., 2018). On the other hand, the deletion of FABP5 in macrophages resulted in increased insulin signaling and glucose uptake in adipocytes. These observations indicate that the interactions between FABP5 and adipocyte/macrophage are critical for the inflammatory basis of metabolic deterioration (Furuhashi et al., 2008).

### The Role and Mechanism of FABP5 in Skin Disease

**FIGURE 7:**
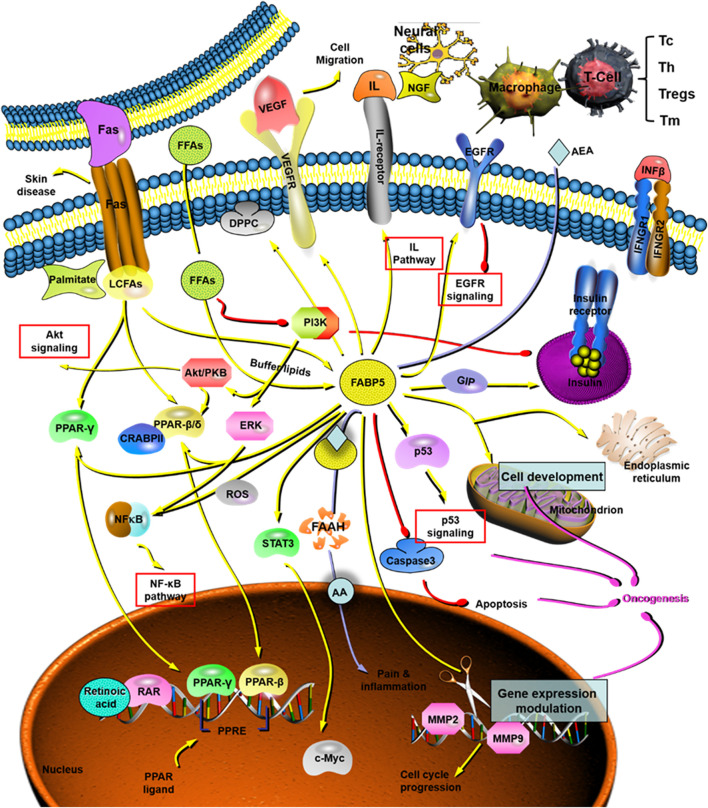
The role and mechanism of FABP5 in tumorigenesis and cancer development. FABP5 plays critical roles in cancer initiation and progression through the AKT pathway, NF-κB pathway, IL pathway, EGFR pathway, p53 pathway, and so on. AA: arachidonic acid; AEA: endocannabinoid anandamide; CRABP: cytosolic retinoic acid-binding protein; DPPC: dipalmitoyl phosphatidyl choline; EGFR: epidermal growth factor receptors; ERK: extracellular regulated protein kinases. FAAH: fatty acid amide hydrolase; FAS: fatty acids; FFA: free fatty acids; FABP5: fatty acid binding protein 5; GIP: glucose-dependent insulinotropic polypeptide; IFN: interferon; IL: interleukin; LCFAS: long-chain fatty acids; MMP: matrix metalloproteinase; NF-κB: nuclear factor-kappa B; NGF: nerve growth factor; PI3K: phophatidylinositol-3-kinase; PPAR: peroxisome proliferator-activated receptor; PPRE: proliferator-activated response element; RAR: retinoic acid receptor; ROS: reactive oxygen species; STAT: signal transducer and activator of transcription; Tc: cytotoxic T cell, CD8^+^ T cell; Th: helper T cell, CD4^+^ T cell; Treg: regulatory T cell; Tm: memory T cell.

The skin is thought to have numerous lipid-rich microenvironments because the epidermis and sebaceous glands are active sites for fatty acids synthesis. Therefore, it is doubtless that FABP5 plays a crucial role in skin homeostasis and pathology (Figure 7). FABP5 is found localized in the suprabasal layers of healthy epidermis, with elevated expression in the spinous, granular layers, and actively proliferating tissue (Ogawa et al., 2011). FABP5 was initially detected and cloned in psoriasis, a hyperproliferative skin disease characterized by abnormal differentiation and disordered lipid metabolism (Madsen et al., 1992). The expression level of FABP5 is markedly elevated in this pathological situation, and it mainly resides in the stratum spinosum (Watanabe et al., 1997). In addition, silencing or overexpressing FABP5 significantly influences keratinocyte differentiation in normal and psoriatic cells, suggesting a link between FABP5 and keratinocyte differentiation (Dallaglio et al., 2013). The lipid composition of epidermal layers is also altered during keratinocyte differentiation, suggesting that FABP5 induces normal human keratinocyte proliferation and differentiation by altering lipid metabolism. Moreover, the up-regulation of FABP5 may be partially involved in the alteration of the differentiation mechanism in psoriasis. In addition to psoriasis, FABP5 is also involved in atopic dermatitis (AD) (Yamane et al., 2009), radiation-induced skin fibrosis (Song et al., 2018), and skin tumor development (Zhang et al., 2018), as a biochemical marker in the skin’s horny layer.

Recent studies have unraveled the function of FABP5 as a prominent marker of sebaceous glands and anagen follicle bulbs (Collins and Watt, 2008). High expression of FABP5 has been consistently found in differentiated sebocytes, while the size of sebaceous glands is significantly reduced in FABP5-deficient mice. Furthermore, the sebum volume was increased as the lipid composition changed, suggesting that FABP5 may regulate the activity of the sebaceous gland through the modulation of cellular lipid signaling and metabolism in the sebocytes (Sugawara et al., 2012). Another study has also reported that FABP5 acts as an antioxidant in the skin by covalently modifying cysteine residues to scavenge peroxidized lipids (Bennaars-Eiden et al., 2002). Additionally, Kusakari et al. (2006) reported an increased expression of FABP5 in regenerative keratinocytes of healing wounds, suggesting that the FABP5 elevation may be warranted in the activation of regenerative epidermal cells motility during wound healing (Kusakari et al., 2006).

Although the function of FABP5 in skin homeostasis and pathology has not been elucidated, it is clear that FABP5 is responsible for the water permeability barrier of the skin. Interestingly, only a minor decrease in the trans-epidermal water loss was observed in the FABP5-deficient mice, and the loss was lower in the knock-out mice than in the wild-type mice (Owada et al., 2002a). However, the skin analyses indicated that there were no differences in the contents of major fatty acids between the knock-out and wild-type mice (Owada et al., 2002b). Considering the robust correlation between FABP5 and FABP3 (H-FABP), a possible explanation might be associated with the compensatory roles that H-FABP plays in the tissues (Owada et al., 2002b). In view of the important role of FABP5 in the skin, further elucidation of the underlying molecular mechanism should be done.

### The Role and Mechanism of FABP5 in Brain Function and Neurological Diseases

Recently, inspired by the essential roles of fatty acids, especially long-chain PUFAs in the normal development of the central nervous system (Liu et al., 2010), the cellular distribution, transcriptional regulation, and function of FABP5 in the normal and diseased brain of humans have been further studied. Studies have reported that FABP5 contributes to neurogenesis and is widely expressed in various cell types (Gerstner et al., 2008), especially mature neurons, in most regions of the brain (Matsumata et al., 2012). The hippocampi of FABP5-null mice had an excessive accumulation of neuronal progenitor cells and a deficit in mature neurons when compared with wild-type animals (Yu et al., 2012). Yu et al. (2014) reported that FABP5 regulates hippocampal function by promoting the hydrolysis of the endocannabinoid anandamide into a form acting as a PPARβ/δ ligand. In addition, FABP5 promotes the hydrolysis of AEA into AA and directly shuttles AA to the nucleus and delivers it to PPARβ/δ, which enables its activation. Mice with FABP5 ablation displayed excess accumulation of AEA and abolishment of PPARβ/δ activation in the brain, thereby significantly impairing hippocampus-based learning and memory. This indicated that FABP5 plays an important role in regulating hippocampal cognitive function by controlling the distribution and activity of anandamide (Yu et al., 2014).

Docosahexaenoic acid (DHA) is an omega-3 PUFA, which serves as a beneficial factor in cognitive function and memory. DHA is sufficiently essential in brain function and its levels are reduced in neurodegenerative diseases such as Alzheimer’s disease (Low et al., 2020). An intracellular carrier protein is required to facilitate the cytosolic trafficking of DHA across the brain endothelial cell to maintain the brain levels of DHA since DHA is not produced by the brain. FABP5 is the key contributor to the uptake of DHA and subsequent trafficking across the blood-brain barrier (BBB) (Pan et al., 2016). In addition, FABP5 is an important player in the promotion of cellular uptake, transport, and metabolism of DHA after spinal cord injury, and thus it has been considered as a significant contributor to basic repair mechanisms in the injured spinal cord (Figueroa et al., 2016). Similarly, a previous study reported that the FABP5 expressed in primary human brain endothelial cells is involved in the transport of palmitic, oleic, and linoleic acids across the primary brain endothelial cells. This finding is consistent with the role of FABP5 in transporting DHA (Mitchell et al., 2011). All the studies conducted on DHA highlight the importance of FABP5 in availing DHA to the brain as an intracellular protein, thereby guaranteeing the growth and functional development of the brain.

As for the role of FABP5 in the pathogenesis of schizophrenia and autism spectrum disorder (ASD), further studies have proved that disturbances in brain-expressed FABP5 could result in a proportion of psychiatric illnesses (Shimamoto et al., 2014). FABP5 is involved in acoustic startle response and performance of prepulse inhibition (PPI), a deficit of which is a biological marker for psychiatric illnesses such as schizophrenia and bipolar disorder (Iwayama et al., 2010). However, mice deficient for FABP5 exhibits no difference in other behavior performances, such as spatial memory, anxiety-like behavior, and diurnal changes of general activity (Matsumata et al., 2016). Moreover, FABP5 expression is up-regulated (or altered) under pathological conditions, such as peripheral nerve injury, ischemic brain damage, and stroke, as well as a neurodegenerative disorder, such as Creutzfeldt-Jakob disease (Lescuyer et al., 2004; Ma D. et al., 2010).

### The Role and Mechanism of FABP5 in Tumorigenesis and Cancer Development

FABP5 is found up-regulated in the tumor-associated epithelial cell, and it affects tumor growth and progression by binding transported fatty acids and their derivatives, hormones, steroids, carcinogens, and other ligands (Zimmerman et al., 2001). In addition, there are several previous studies on the correlation between fatty acids and tumors. It is common knowledge that various types of cancers display increased biosynthesis of endogenous fatty acids, whereas the *de novo* synthesis of fatty acids is inhibited in most normal cells except for adipocytes and hepatocytes (Mashima et al., 2009). For example, prostate cancer utilizes fatty acid oxidation as the main bioenergy pathway for supporting proliferation (Zadra et al., 2013). The rationale behind this phenomenon is that the survival of cancer cells depends on the uptake and consumption of fatty acids to maintain their rapid proliferation and metastatic behavior, which further leads to cancer initiation, progression, and metastasis (Daniëls et al., 2014). Silencing or down-regulating FABP5 in human cancer cells inhibits cell proliferation and decreases the expression of the genes which are involved in altered lipid metabolism, lipolysis, and *de novo* synthesis of fatty acids in various tumors. Therefore, it is possible for FABP5 to act as a potential biomarker for tumors based on the key role of FABP5 in lipid metabolism, especially fatty acids metabolism.

Neoplastic transformation and tumor growth, two fundamental processes in early tumorigenesis, are characterized by acquiring the traits of sustained proliferation and cell death resistance (Hanahan and Weinberg, 2011). A comparison of normal cells with benign hyperplasia indicates that FABP5 is ubiquitously overexpressed in most the cancers including breast cancer, cervical cancer (CCa), endometrial cancer (EC), head and neck squamous cell carcinoma (HNSC), prostate cancer (PCa), hepatocellular carcinoma (HCC), tongue carcinoma, esophageal cancer (ESCA), clear cell renal cell carcinoma (ccRCC), stomach adenocarcinoma (STAD), and lung squamous cell carcinoma (LUSC) **(** Table 2). Mechanism studies have revealed that FABP5 regulates tumorigenesis and cancer progression by regulating different pathways including MMP-2/MMP-9, PI3K/AKT, and PPARs pathways (Rauch et al., 2004; Shi et al., 2012; Forootan et al., 2014; Ohyama et al., 2014; Nitschke et al., 2020) (Figure 7 and Table 2). Ki67, a notable cell proliferation marker in the tumor, could independently predict cancer progression (Yang et al., 2018). It was reported that the number of Ki67-stained cells was significantly decreased in the FABP5 knockdown group and significantly increased in the FABP5 overexpression group (Zhang et al., 2020) (Table 2). In addition, silencing FABP5 may reduce the invasiveness of cancer cells, inhibit cell proliferation, and increase cell apoptosis (Powell et al., 2015; Zhao et al., 2017; Lv et al., 2019) (Table 2). All these strong pieces of evidence prove that FABP5 is an important regulator of tumor cell proliferation.

**TABLE 2:** The roles and mechanisms of FABP5 in various types of cancer.

Cancer	Biological function	Mechanism	Refs
Gastric cancer	Promotion	FABP-5 gene silencing inhibit cell proliferation, and arrest cell cycle in G0/G1 phase	Zhao et al. (2017)
Breast cancer	Suppression	Regulate IFN-β responses in TAMs to enhance the effects of macrophage immune surveillance	Zhang et al. (2014)
Breast cancer	Promotion	Activate EGFR and enhance the ability of PPARδ to promote oncogenic properties	Levi et al. (2013)
Breast cancer	Promotion	Upregulate the expression of the VEGF gene	Jing et al. (2000)
Triple negative breast cancer	Promotion	Stabilize EGFR protein expression by preventing EGFR ubiquitination and degradation leading to metastasis	Powell et al. (2015)
Triple negative breast cancer	Promotion	Promote cell survival through the RA-FABP5-PPARβ pathway	Liu et al. (2011)
Cervical cancer	Promotion	Reprogram FA metabolism to activate NF-κB signaling, leading to promotion of EMT, lymphangiogenesis and LNM	Zhang et al. (2020)
Cervical cancer	Promotion	Promote carcinogenesis and metastasis *via* up-regulating MMP-2 and MMP-9	Zhan et al. (2019)
Cervical cancer	Promotion	Inhibit MMP-2 and MMP-9 expression *in vivo* and *in vitro*	Wang et al. (2016)
Clear cell renal cell carcinoma	Promotion	Mediate signaling pathways involved in EMT	Wu et al. (2019)
Clear cell renal cell carcinoma	Promotion	Regulate the proliferation *via* the PI3K/AKT signaling pathway	Lv et al. (2019)
Squamous cell carcinoma	Promotion	Rapidly upregulated upon induction of EpCAM as a major target of c-Myc	Uma et al. (2007)
Hepatocellular carcinoma	Promotion	Promote angiogenesis and activate the IL6/STAT3/VEGFA pathway	Pan et al. (2018)
Hepatocellular carcinoma	Promotion	Induce EMT	Ohata et al. (2017)
Intrahepatic cholangiocarcinoma	Promotion	Increase mobilization of fatty acids	Jeong et al. (2012)
Prostate cancer	Promotion	Transport excessive amounts of FA into the nucleus to stimulate PPARγ leading to a reduced apoptosis and an increased angiogenesis	Naeem et al. (2019)
Prostate cancer	Promotion	FABP5/PPARβ/δ pathway	Morgan et al. (2010)
Prostate cancer	Promotion	Hypomethylation of FABP5 promoter leads to the up-regulation of FABP5 transcription by Sp1 and c-Myc	Kawaguchi et al. (2016a)
Prostate cancer	Promotion	Interact with PPARγ in a coordinated mechanism to facilitate malignant progression	Forootan et al. (2014)
Prostate cancer	Promotion	Activate PPARγ and estrogen-related receptor α	Carbonetti et al. (2019)
Prostate cancer	Promotion	Suppression of FABP5 reduces the expression of VEGF and thus inhibit the tumorigenicity	Adamson et al. (2003)
Prostate cancer	Promotion	activate PPARγ and upregulate the expression of VEGF.	Bao et al. (2013)
Colorectal cancer	Promotion	Promote cell growth and invasion by a PPARβ/δ-independent signaling pathway	Kawaguchi et al. (2016b)
Oral squamous cell carcinomas	Promotion	Increase the expression of MMP-9	Fang et al. (2010)
Skin tumor	Suppression	Regulate IFN/p53/SOX2 pathway to suppress skin tumorigenesis	Zhang et al. (2018)

Epithelial-mesenchymal transitions (EMT) is a multistep morphogenetic process in which epithelial cells lose their epithelial properties while fully acquiring their mesenchymal properties. It is thought to be a critical process in the migration, invasion, and metastatic spread of cancer cells (Thiery and Sleeman, 2006). It was reported that FABP5 may regulate the development and progression of cancer cells by mediating signaling pathways that are involved in EMT (Wu et al., 2019) (Table 2). Another study reported that the overexpression of FABP5 in oral cancer cells enhances cell proliferation and invasiveness by increasing the expression of MMP-9, while the knockdown of FABP5 using shRNA significantly inhibits the activity of MMP-9 (Fang et al., 2010). These findings suggest an undeniable correlation between FABP5 and MMP-9 (Table 2). Similarly, the down-regulation of FABP5 in cervical cancer inhibits the *in vivo* and *in vitro* expression of MMP-2 and MMP-9 (Wang et al., 2016) (Table 2). In the mechanism, the extracellular matrix is degraded and the basal membrane expression of cervical cancer cells is destroyed by up-regulating the expression of MMP-2 and MMP-9, thereby resulting in carcinogenesis and metastasis (Zhan et al., 2019) (Table 2). Furthermore, FABP5 (as an epithelial marker) can be inhibited by autophagy and thus induce the expression of mesenchymal markers as well as MMP-9, thereby stimulating cell invasion (Li et al., 2013). Collectively, great progress has been made in revealing the interactions among FABP5, MMP-9, and EMT. However, definitive evidence on the underlying molecular mechanism and signaling pathway have not been provided.

Interestingly, some studies have exact opposite results. FABP5 has been shown to suppress chemically-induced skin tumorigenesis through regulation of the IFN/p53/SOX2 pathway, where it down-regulates the expression of SOX2 mediated by p53 in keratinocytes (Figure 7). Herein, FABP5 represents a previously unknown molecular mechanism where the host maintains skin homeostasis thereby preventing the skin tumorigenesis induced by environmental insults (Zhang et al., 2018) (Table 2). Hammamieh, et al. (2004) reported that FABP5 was down-regulated in prostate cancer cell lines (DU145) and breast cancer cell lines (MCF-7) when compared with that of normal cells in tissue cultures and biopsy samples (Hammamieh et al., 2004; Hammamieh et al., 2005). And it was also found that the expression of FABP5 in different types of human breast cancer tissues was significantly lower than that of normal breast tissues (Zhang et al., 2014). Recent studies have helped us to further unravel the underlying mechanisms. One possible explanation is that the regulation of FABP5 in intracellular immune responses enhances tumor immune surveillance and induces tumoricidal activity. However, the peculiarities of cancer metabolism might counteract the immune killing effect of FABP5 on a tumor, which may partially explain why FABP5 is down-regulated in PCa and breast cancer. Specifically, PCa and breast cancer presents unique metabolic features as they use lipids as the dominant energy source while general cancers are characterized by an accelerated glucose utilization. (Slebe et al., 2016). This can probably be attributed to the metabolic adaptation to lipid-rich and/or glucose-poor cellular microenvironments. Future studies should aim at further exploring the exact roles of FABP5 in anti-tumor immunological surveillance or in promoting tumor progression through lipid metabolism, and the balance between them.

FABP5/PPARβ/δ pathway is critical in tumorigenesis and cancer growth (Figure 7), which is similar to its role in enhancing the proliferation of keratinocytes (Schug et al., 2007; Morgan et al., 2010) (Table 2). FABP5 regulates metabolic gene expression through a PPARβ/δ independent pathway in colorectal cancer cells (Kawaguchi et al., 2016b) (Figure 7). However, there is a discrepancy where some literature has reported that PPARβ/δ signaling does not enhance the growth of human cancer cell lines and it attenuates colon carcinogenesis (Marin et al., 2006; Hollingshead et al., 2007; Palkar et al., 2010). Similarly, another study reported that PPARβ/δ is not a potential biomarker for assessing the degree of malignancy or a prognosis predictor of PCa because there was no correlation found between the expression of PPARβ/δ and patient survival (Forootan et al., 2014) (Table 2).

In addition to its important role in tumorigenesis, FABP5 is also closely correlated with tumor grade and metastasis (Liu et al., 2013). The alteration of lipid metabolism plays a crucial role in cancer development and metastasis. For instance, the dysregulation of lipid metabolism and signaling pathway has been recognized as a major driver of PCa metastasis (Ahmad et al., 2016; Zadra et al., 2019). Moreover, high levels of lipid droplets and stored cholesteryl ester contents are the hallmarks of cancer aggressiveness (Bozza and Viola, 2010). Therefore, FABP5 might be implicated in cancer metastasis through the reprogramming of lipid metabolism. FABP5 is involved in the malignant dissemination of some human cancers (Jing et al., 2000) (Table 2). For instance, Jing et al. (2001) transfected a benign, non-metastatic rat mammary epithelial cell line (Rama 37 cells) with FABP5 and then inoculated it in syngeneic rats. Almost one-third of FABP5-transfected animals developed metastases, while the animals treated with non-FABP5-transfected remained free of metastases. This indicates that an elevated expression of FABP5 may induce metastasis of cancer cells (Jing et al., 2001). In addition, FABP5 has been identified as an independent predictor of lymph node metastasis (LNM) (Jeong et al., 2012; Wang et al., 2014). The mRNA and protein expression levels of FABP5 in cervical cancer (CCa) cells derived from lymph node metastatic sites (MS751) were significantly higher than those in CCa cells derived from primary sites (HeLa and SiHa) (Zhang et al., 2020). FABP5 was also highly expressed in invasive cancer cell lines (LI-7 and HLE) than in the noninvasive cell lines (Hep3B and HepG2) of HCC (Ohata et al., 2017) (Table 2). This finding confirmed the role of FABP5 as a promoter of tumor metastasis. Nevertheless, there was no notable difference in the expression of FABP5 between cancer tissues with lower Gleason scores (scores of 1–5) and those with higher Gleason scores (scores of 5–10). This suggested that FABP5 does not increase with the increasing metastatic potential and that FABP5 may play a more important role in the initiation of early malignant transformation than in the promotion of metastasis (Adamson et al., 2003) (Table 2).

Overexpression of FABP5 interacts with PPARγ in a coordinated manner to promote malignant progression of tumor including tumor expansion and aggressiveness caused by reduced apoptosis and increased angiogenesis (Naeem et al., 2019) (Table 2). In addition to PPARγ, other receptors including the related PPARβ/δ and estrogen-related receptor α, and signaling involving VEGF are also known to increase the metastatic potential of cancer (Carbonetti et al., 2019) (Table 2). As mentioned before, FABP5 affects the survival pathways by activating RA through PPARβ/δ, and the ratio of FABP5 to CRABPII may lead to the results of natural apoptosis, or unrestricted cell growth (Schug et al., 2007; Zhou et al., 2016).

Despite the molecular cascades of metastasis involving polygenic and complex alterations, angiogenesis is still an important common mechanism for metastasis and the formation of solid tumors. As discussed above, VEGF has been identified as a key mediator of angiogenesis in cancer and FABP5 has been positively correlated with VEGF expression (Figure 7). Studies have revealed that the microvascular density of metastatic tumors and their primary tumors transfected with FABP5 is significantly higher than that of the primary tumors developed from the control vector transfectants (Jing et al., 2000). However, the effect of FABP5 on VEGF is limited since a further increase in FABP5 may have a little additional effect on the expression of VEGF once the threshold level is exceeded, which might explain why FABP5 and VEGF are not under a simple linear relationship. FABP5 was demonstrated to promote angiogenesis through activating the IL6/STAT3/VEGFA pathway in HCC (Pan et al., 2018) (Table 2), or transporting excessive levels of fatty acids into the nucleus of the cancer cells to activate PPARγ thereby up-regulating the expression of VEGF (Bao et al., 2013) (Figure 7 and Table 2). Moreover, FABP5 can induce the production of reactive oxygen species (ROS) and activate nuclear factor-kappa B (NF-κB) signaling, which leads to the up-regulation of its target genes (Senga et al., 2018) (Figure 7). In addition, NF-κB in turn induces the expression of FABP5, by activation of epidermal growth factor receptors (EGFR) ligand heregulin-β1 signals through the ERK and the phophatidylinositol-3-kinase cascades (Kannan-Thulasiraman et al., 2010) (Figure 7). Mechanistic studies have also revealed that FABP5 stabilizes EGFR protein expression by preventing EGFR ubiquitination and degradation, thereby promoting metastasis. This indicates its special effect on EGF-induced metastatic signaling (Powell et al., 2015) (Table 2). FABP5 was also found as a direct target of c-Myc, a proto-oncogene, leading to the up-regulation of FABP5 transcription through the hypo-methylation of the FABP5 promoter (Uma et al., 2007; Kawaguchi et al., 2016a) (Table 2). And it was confirmed in squamous cell carcinoma (SCC) lines and primary head and neck carcinomas (Münz et al., 2005).

## Conclusion and Future Perspectives

FABP5 was initially thought to be a transfer protein for cellular lipid but it is currently recognized as a key molecule in the pathogenesis of over nutrition-related diseases, and a modulator of cytokine production and signaling transduction in select cell types and tissues. This review has summarized the current knowledge on the structural organization and tissue localization of this specific protein. We have discussed its biological properties, canonical and newly postulated physiological functions, and roles in diseases.

Accumulating evidence has confirmed that FABP5 plays an essential role in cellular FA transport and utilization, and it has been indirectly implicated in FA-mediated regulation of gene expression, partly through PPARs. Previous studies have also revealed the function of FABP5 in regulating intracellular fatty-acid configuration and how these alterations are correlated with specific biochemical pathways involved in metabolic homeostasis. As the functions of FABP5 are being further elucidated, it has become evident that FABP5 is central to lipid-mediated and related metabolic processes through the utilization of various genetic and chemical models both locally and systemically. Moreover, therapeutic FABP5 research has focused on animal models of obesity, diabetes mellitus, and cardiovascular diseases with the obtained results indicating an association between FABP5 levels and disease risk and adverse prognosis. More importantly, it can be assumed that FABP5 will be used in clinical work for the diagnosis of metabolic syndrome and cardiovascular diseases in the future based on the studies surrounding the clinical applications of FABP5. With regards to therapeutic applications, FABP4/5 inhibitors have been shown to ameliorate dyslipidemia in diet-induced obesity mice. This has highlighted their great potential as therapeutic targets for a range of associated disorders including obesity, diabetes, and atherosclerosis.

Lipid metabolism is an essential and complex physiological process for organisms, which ensures normal physiology and is of great significance for life activities. Dysregulation of lipid metabolism creates conditions for various diseases, including metabolic diseases, neurocognitive deficits, autoimmune diseases, cancer, COVID-19, etc. (Seo et al., 2020; Tanner and Alfieri, 2021). FABP5 are involved in regulating lipid metabolism at multiple steps, therefore play a critical role in the pathogenesis of several lipid metabolism-related disorders. Lipid metabolism reprogramming is an emerging hallmark of malignancy (Cheng et al., 2018; Bian et al., 2021). Exploring the mechanisms by which FABP5 is involved in tumors from the perspective of lipid metabolism can be a valuable research direction that requires further exploratory studies. Meanwhile, there are still interesting contradictions that remain to be fully explained, in terms of, for example, lipolysis and PPAR activity. A complete understanding of mechanisms by which FABP5 is involved in lipid metabolism will address the barriers to treating a wide variety of diseases by targeting altered lipid metabolism (Cao, 2019; Cheng et al., 2019; Sangineto et al., 2020).

It is also worth noting that, FABP5-deficient mice are viable and show no macroscopical aberrations when compared with wild-type mice, which indicates that the lack of FABP5 is not fatal. One possible explanation from the overview of the current mechanism studies is that the functions of FABP5 may be partly compensated by additional FABP family members or other proteins, which is similar to the observation in several studies of up-regulated FABP3 expression when FABP5 is knocked out (Owada et al., 2002b). Consequently, it is one of the most important areas in future research because it will resolve the issues of how different FABPs interact with each other and the biological effects of these interactions.

In addition, the link of FABP5 with evolution and nutritional conditions has emerged as a novel focus in recent years. Several researchers have found that FABP5 plays a key role in thermogenesis under fasting and cold stress conditions, thereby underscoring the significance of FABP5 in overcoming life-threatening conditions such as cold and starvation (Syamsunarno et al., 2014). Another study conducted on fasted mice reported that FABP5 has an essential role in maintaining exercise endurance capacity in nutrient homeostasis during prolonged fasting (Iso et al., 2019). Therefore, FABP5 may play a crucial role in regulating adipocyte function to deal with the changes in nutritional conditions.

FABP5 has been found to be substantially expressed in the skin and in immunocytes, especially macrophages, when compared with other FABPs. The interactions of FABP5 with APC and T cells, and its role in thymic immunity suggest its unique and non-negligible role in human immune function such as the host defense against infection and regulation of acute inflammatory response. FABP5 might also modulate the activation of monocytes activation and may be a promising target for therapeutic intervention after allograft rejection. Moreover, recent studies have proposed FABP5 as an intracellular transporter of AEA, which is closely associated with analgesic action. FABP5 is found highly expressed in nociceptive ganglia neurons and FABP5 inhibitors exhibit peripheral and supraspinal analgesic effects (Peng et al., 2017). These novel observations suggest that FABP5 is a promising target for the development of inhibitors to help control pain and inflammation.

The role and mechanism for FABP5 in cancer initiation and progression have been gradually identified since the last decade. FABP5 is not a “spectator,” but a positive “participant” in the whole process of tumorigenesis, development, and metastasis. Cytoplasmic FABP5 has also been proved as a significant and independent prognostic marker of overall survival and recurrence-free survival in cancers. Although expressed in almost all types of cancer cells, FABP5 is a double-edged sword for cancer: overexpression of FABP5 leads to tumorigenesis and metastasis while selective enhancement of FABP5 activity in macrophages may promote immune surveillance toward tumor and thus inhibit tumor progression. Inspired by the interactions between FABP5 and RA/PPARs (Figure 7), a model whereby growth-promoting FABP5 competes with growth-inhibiting CRABP2 for RA has been proposed. Retain RA in the cytoplasm by FABP5 to prevent tumor growth (Liu et al., 2011). Based on such studies, several substances (e.g., curcumin) have been utilized for suppression of the FABP5/PPARβ/δ pathway and thereby inhibit cancer cell growth (Thulasiraman et al., 2014).

The regulators or inhibitors of the FABP5 may have therapeutic potential in cancer. Here is a notable example. The growth and expansion of prostate cancer depend on androgen stimulation in the early stage. During androgen-deprivation therapy (ADT), the androgen receptor (AR)-initiated pathway is suppressed, only a small percentage of cancer cells survived as they have successfully switched energy sources to fatty acids. As the degree of malignancy or androgen independency increases, The FABP5-related pathway is gradually replacing the AR-initiated pathway as the predominant pathway in AR-negative, androgen-independent castration-resistant prostate cancer (CRPC) cells. Thus, the inhibition of FABP5-related signaling pathways, which is entirely distinct from the existing androgen-blocking-based treatments, is essential for the suppression of the malignant progression of CRPC cells (Al-Jameel et al., 2017). And it will be rather meaningful to figure out whether inhibiting the FABP5-related pathway can also suppress the progression of other FABP5-positive cancers. Meanwhile, it was found that FABP5 inhibitors can enhance the cytotoxicity and tumor-suppressive effects of chemotherapy drugs (e.g., taxanes) on cancer cells, and may also potentially decrease drug resistance (Carbonetti et al., 2020). To conclude, the critical role of FABP5 in tumorigenesis and progression provides new possibilities for the early diagnosis of tumors and a novel strategy for tumor prevention and treatment.

The role of FABP5 in normal and pathological growth and cell signaling is also worthy of attention. In addition to directly targeting molecules reviewed above, the functions of FABP5 to regulate lipid signaling and transport in a tissue-specific or restrictive manner can also be exploited to control the activity of its targets, such as nuclear hormone receptors in select cell types and tissues. In general, two main mechanisms for FABP5 function are related to direct ligand binding and transport within the cytoplasmic compartment, and interactions with nuclear hormone receptors to regulate downstream processes, including signal transduction cascades and intermediary metabolic pathways. Therefore, it can be expected that the creation of pharmacological agents leading to the modification of the FABP5 function may provide tissue-specific or cell-type-specific control of lipid signaling pathways, inflammatory response, and metabolic regulation, thus providing a new class of multi-indications for treatment (Furuhashi et al., 2011). However, in light of the dual biological effects of FABP5 in various aspects, identifying the distinction and correlation between the negative and positive impacts is of utmost importance. Prior to that, it is supposed to figure out the mechanism underlying the reconciliation of the opposing effects of FABP5. Equally important is the identification of both inducers and inhibitors of FABP5 expression in future studies as they might have potential therapeutic applications.

The research progress has led to some complexities arising from the need to elucidate the underlying mechanisms of FABP5, and the need has grown exponentially with the advancement of the research on biological functions of FABP5. The complexities can be briefly summarized as follows: 1) the interaction of FABP5 with membrane lipids, plasma membrane FA transporters, intracellular membranes FA acceptor proteins, and FA metabolic enzyme systems; 2) interactions of FABP5 with different ligands and corresponding downstream cascades, and the molecules and signals involved; and 3) the exact central nervous system (CNS) functions that rely on FABP5 since only limited information is available for its role in the brain and their involvement in neurological diseases. Unraveling these details will require a concerted effort, but they also provide an exciting opportunity to develop novel therapeutic approaches.
